# Whey Protein Concentrate as a Novel Source of Bifunctional Peptides with Angiotensin-I Converting Enzyme Inhibitory and Antioxidant Properties: RSM Study

**DOI:** 10.3390/foods9010064

**Published:** 2020-01-08

**Authors:** Fatima Abdelhameed Hussein, Shyan Yea Chay, Mohammad Zarei, Shehu Muhammad Auwal, Azizah Abdul Hamid, Wan Zunairah Wan Ibadullah, Nazamid Saari

**Affiliations:** 1 Department of Food Science, Faculty of Food Science and Technology, University Putra Malaysia, Serdang, Selangor 43400, Malaysia; fatima_abdelhameed@yahoo.com (F.A.H.); shyan_yea@upm.edu.my (S.Y.C.); azizahah@upm.edu.my (A.A.H.); wanzunairah@upm.edu.my (W.Z.W.I.); 2 Department of Dairy Production, Faculty of Animal Production, University of Khartoum, PO Box 32, Khartoum North 13314, Sudan; 3 Department of Food Science and Technology, Faculty of Applied Sciences, Universiti Teknologi MARA, Shah Alam, Selangor 40450, Malaysia; mzarei.mail@gmail.com; 4 Department of Biochemistry, Faculty of Basic Medical Sciences, Bayero University, Kano 700231, Nigeria; samuhammad.bch@buk.edu.ng

**Keywords:** ACE inhibition, antioxidant activity, hydrolysis, response surface methodology, whey protein concentrate

## Abstract

Whey protein concentrate (WPC) is a unique source of protein with numerous nutritional and functional values due to the high content of branched-chain amino acid. This study was designed to establish the optimum conditions for Alcalase-hydrolysis of WPC to produce protein hydrolysates with dual biofunctionalities of angiotensin-I converting enzyme (ACE) inhibitory and antioxidant activities via response surface methodology (RSM). The results showed that the optimum conditions were achieved at temperature = 58.2 °C, E/S ratio = 2.5%, pH = 7.5 and hydrolysis time = 361.8 min in order to obtain the maximum DH (89.2%), ACE-inhibition (98.4%), DPPH• radical scavenging activity (50.1%) and ferrous ion chelation (73.1%). The well-fitted experimental data to predicted data further validates the regression model adequacy. Current study demonstrates the potential of WPC to generate bifunctional hydrolysates with ACE inhibition and antioxidant activity. This finding fosters the use of WPC hydrolysate as a novel, natural ingredient for the development of functional food products.

## 1. Introduction

Hypertension is considered a key risk factor in cardiovascular-related diseases including stroke, peripheral and coronary heart diseases. The total number of adults who had elevated blood pressure was 594 million in 1975 and it increased tremendously to 1.13 billion in 2015 [1], attributed to unhealthy diets and poor lifestyle such as alcohol intake and smoking. By 2025, it is projected that hypertension will affect more than 1.5 billion people globally [2]. Angiotensin-I converting enzyme (ACE) has been identified as the key enzyme which increases blood pressure in human body. It cleaves the inactive decapeptide, angiotensin-I, to an octapeptide, angiotensin-II, which acts as a potent vasoconstrictor, as well as inactivates the vasodilating nonapeptide, bradykinin. The dual action of ACE results in a rise in the blood pressure. Inhibition of ACE by reducing angiotensin II generation and increasing bradykinin generation is necessary to control high blood pressure. In recent years, the increasing health awareness drives consumers to look for natural alternatives that are safer with minimal side effects compared to synthetic blood pressure-controlling drugs. Interestingly, positive results have been garnered with the discovery of bioactive peptides derived from various food proteins.

Whey proteins concentrate (WPC), being a natural valuable peptide source, provides considerable nutritional and health benefits for humans due to its high level of branched-chain amino acids, high protein content (30–80%) [3,4,5] and presence of promising functional molecules including immunoglobulin, α-lactalbumin, lactoperoxidase, albumin, lactoferrin and caseinomacropeptide [6,7]. However, WPC is less studied as compared to whey protein (the crude, non-purified form) and whey protein isolate (higher protein content than WPC). Hydrolysis of these whey products would generate smaller peptide fragments with different bioactivities, such as antioxidative [8], antihypertensive [9,10,11], immunomodulatory [12], antithrombotic [13], antimicrobial [14] and opiate properties [15]. Of all these bioactivities, ACE inhibition and antioxidant activity are extensively studied, but mainly on a separate basis. For instance, Guo, Pan and Tanokura [9] and O’keeffe, Conesa and FitzGerald [16] reported ACE inhibition between 40.0–84.4% for different whey products while Ajlouni and Pan [17] as well as Zhang et al. [18] reported antioxidant activities between 0.0–72.1%, respectively.

Hydrolysis parameters rely heavily on the purpose of hydrolysis. Different purposes would yield different sets of optimum conditions and enzyme selection. WPC has been widely used to evaluate the optimum hydrolysis conditions using different enzymes. For instance, Guo, Pan and Tanokura [9] reported the optimum temperature = 38.9 °C, pH = 9.2 and E/S ratio = 0.60 for *L.* helveticus LB13 crude proteinase hydrolysis while Naik et al. [19] reported the optimum temperature = 35.5 °C, time = 8 h and pH = 7.3 when Flavourzyme was used. Also, WPC has been hydrolysed using Alcalase, the same enzyme in current work, in several other studies. According to Athira et al. [20], the optimum conditions for Alcalase-assisted WPC hydrolysis were 55 °C, 8 h and pH 9 while Fenoglio et al. [21] suggested to use 50 °C, pH 8–9 and 25 min to achieve optimum hydrolysis. While these studies used the same substrate and same enzyme as in current work, they produced different sets of optimum temperatures, pH and hydrolysis time, depending on the different objectives (i.e., the measured responses) targeted by different works. For instance, Athira et al. [20] focused on single bioactivity of WPC, Fenoglio et al. [21] measured the production of bitter peptides for dessert making while current work measured multiple bioactivities from WPC.

Unlike most works that focused on single bioactivity of WPC, current study evaluates the dual functionality of WPC hydrolysate in terms of ACE inhibition and antioxidant activities by employing 4-factors design using response surface methodology (RSM). RSM is a collection of statistical methods for building a model by setting a combination of factor levels to get an optimum response, by employing a polynomial equation to evaluate the relationship between factors as well as their key and combined effect to induce a desired response [22]. This study serves to determine the optimized conditions to generate WPC hydrolysates that possess maximum ACE-inhibition and antioxidant activities by employing response surface methodology according to Box-Behnken design.

## 2. Materials and Methods

### 2.1. Materials

Whey protein concentrate (WPC) was purchased from Purefit Company (Selangor, Malaysia). ACE from rabbit lung, N-Hippuryl-His-Leu hydrate powder and O-phthaldialdehyde (OPA) were obtained from Sigma Chemical Co., (St Louis, MO, USA). Alcalase was purchased from Novozymes (Copenhagen, Denmark). All other chemicals employed were of analytical grade and obtained from either Acros Organics (Geel, Belgium), Merck KGaA (Darmstadt, Germany), Fisher Scientific (Waltham, MA, USA) or J.T. Baker (Phillipsburg, NJ, USA).

### 2.2. Proximate Analysis of WPC

The proximate analysis was determined according to the method of Association of Official Analytical Chemists [23]. Oven method was employed to measure the moisture content using a forced draft oven (model: Quimis Q-314M242, serial 020, São Paulo, Brazil) at 105 °C until reaching a constant weight. Ash was determined via incineration with a muffle furnace at 550 °C. Soxhlet extraction method with ethylic ether was employed to determine the fat content. The micro Kjeldahl method was used to determine the protein content, whereby a conversion of 6.38 was multiplied. All analyses were performed in triplicates.

### 2.3. Sodium Dodecyl Sulphate Polyacrylamide Gel Electrophoresis (SDS-PAGE)

SDS-PAGE was performed on WPC hydrolysate as described by Laemmli [24], using 40% acrylamide for protein separation. The hydrolysates were adjusted to a 10 mg protein/mL concentration. The sample solutions and the sample buffer (0.5 M Tris-HCl, pH 6.8, containing 4% SDS, 20% glycerol) were mixed at a 3:1 (*v/v*) ratio and heated for 10 min at 95 °C, prior to loading. The concentrations of stacking and resolving gels were 4% and 12%, respectively. The gel was run at 150 V. Aliquots of 10 μL were loaded into individual wells and a constant current was passed through the gel for 1 h to complete the peptide separation. The gel was stained using coomassie blue (5.0 g coomassie brilliant blue R-250 mixed with 500 mL ethanol, 100 mL acetic acid and 400 mL deionized water), distained with distaining solution containing 200 mL of 40% ethanol and 80 mL of 6% acetic acid with 720 mL deionized water, then preserved in 5% acetic acid. The molecular weights were determined by approximation using low range molecular weight standards (Bio-Rad, Hercules, CA, USA).

### 2.4. Response Surface Methodology (RSM) Design for WPC Hydrolysis

While optimum hydrolysis conditions are recommended by the enzyme manufacturer, these parameters are given in ranges without pinpointing exact values. Optimum conditions are subjective and multifactor-dependent (purpose of study, sample nature and equipment availability). Thus, optimum conditions should be specifically considered on a case-by-case basis, justifying the need to perform optimization study on WPC hydrolysis. Box-Behnken design (BBD) was employed for optimisation of WPC hydrolysis parameters, whereby each parameter had three levels coded as −1, 0, +1, based on four independent variables: temperature (°C, x_1_), enzyme to substrate ratio (E/S, x_2_), pH (x_3_) and time (min, x_4_). BBD was chosen over central composite design because it is more economic and suitable for current study which manipulates four independent variables and four dependent variables (responses). A total of twenty-seven runs with three replicates of the central point was performed. For each treatment, the mean pertaining to triplicate measurements was considered as responses for degree of hydrolysis (%, y_1_), ACE-inhibition (%, y_2_), DPPH• (%, y_3_) and ferrous ion chelating (%, y_4_). Randomisation of experimental runs was performed to mitigate the impacts of unexpected variability to actual responses.

Briefly, Alcalase was mixed with WPC in 100 mL borate buffer at different ranges of temperatures (40–70 °C), enzyme/substrate (E/S) ratios (0.5%–2.5%), pHs (pH 6.5–10) and times (30–480 min), then incubated in a water bath shaker at an agitation rate of 150 rpm. At the end of hydrolysis, the mixture was placed in boiling water (100 °C) for 10 min to inactivate Alcalase. Upon cooling, centrifugation at 10,000 *g* was performed for 15 min. The supernatant was stored at −20 °C prior to analysis of degree of hydrolysis (DH), biological activities including ACE-inhibition, DPPH•, and ferrous ions chelating activity.

For data analysis, analysis of variance (ANOVA) and regression equation were employed to determine the regression coefficients, statistical significance of the model terms and to fit the predicted mathematical models with that of the experimental data. The least-squares technique was employed to determine the multiple regression coefficients for predicting quadratic and linear polynomial models pertaining to the calculated response variables [25,26]. The general polynomial model for determining the response is given as follows (Equation (1)):

y = β_0_ + β_1_ x_1_ + β_2_ x_2_ + β_3_ x_3_ + β_4_ x_4_ + β_11_ x_1_^2^ + β_22_ x_2_^2^ + β_33_ x_3_^2^ + β_44_ x_4_^2^ +β_12_ x_1_ x_2_ +β_13_ x_1_ x_3_ + β_14_ x_1_ x_4_ + β_23_ x_2_ x_3_ + β_24_ x_2_ x_4_ + β_34_ x_3_ x_4_ (1)

whereby, y represents the response; *β_0_* signifies the offset term for the design; β_1_, β_2_, β_3_ and β_4_ denote the regression coefficients defining the linear effect terms; β_11_, β_22_, β_33_ and β_44_ signify the quadratic effects; β_12_, β_13_, β_14_, β_23_, β_24_ and β_34_ represent the interaction effects; and x_1_, x_2_, x_3_, and x_4_ represent independent factors in this model.

All linear effects, whether significant or not, are included in the original regression equations (non-reduced model). On the other hand, for square and interaction effects, only significant terms were taken into consideration for reduced polynomial regression pertaining to degree of hydrolysis (y_1_, Equation (2)), ACE-inhibition (y_2_, Equation (3)), DPPH• radical scavenging (y_3_, Equation (4)) and ferrous ion chelating activity (y_4_, Equation (5)).

y_1_ = −116.761 + 3.421x_1_ + 55.453x_2_ − 6.253x_3_ + 0.219x_4_ − 0.056x_1_ x_1_ + 0.36x_1_ x_3_ − 5.603x_2_ x_3_ (2)

y_2_ = −57.8367 − 0.943x_1_ + 33.1444x_2_ + 34.294x_3_ + 0.0542x_4_ − 0.0286x_1_^2^ − 3.347x_3_^2^ − 0.0002x_4_^2^ + 0.4761x_1_ x_3_ + 0.0014x_1_ x_4_ − 4.0186x_2_ x_3_ (3)

y_3_ = −190.337 +6.167x_1_ − 0.313x_2_ + 5.799x_3_ + 0.158x_4_ − 0.050x_1_ x_1_ − 0.018x_3_ x_4_ (4)

y_4_ = −31.8992 + 1.9866x_1_ − 5.3641x_2_ + 13.9083x_3_ + 0.0609x_4_ − 0.0174x_1_ x_1_ − 0.9596x_3_ x_3_ − 0.0002x_4_ x_4_ + 0.0157x_2_ x_4_ (5)

For each response (y-value), four important elements, i.e., *R*^2^, *R*^2^-adj, Fisher values (*f*-values) and *p*-values for regression were taken into account to determine the adequacy of the models in predicting experimental values. Based on reduced models, all significant (*p* < 0.05) interaction effects from two independent factors were visualized as three dimensional (3D) surface plots, with the responses (DH, ACE-inhibitory activity, DPPH• radical scavenging and ferrous ion chelating) plotted on y-axis. Drawing of the 3D plots was obtained by maintaining two constant variables at the centre point while modifying the other two variables within the experimental range. The Minitab statistical package (Minitab Inc., State College, PA, USA) was employed for data analysis, optimisation procedure and experimental design conditions. Specifically, for mathematical optimisation, response optimiser was applied to calculate specific optimum levels of concurrent and individual multiple response optimisations, resulting in appropriate response objectives. The experimental data was compared with the predicted values obtained from the equations to test the adequacy of regression equations. Significant differences were identified at *p* < 0.05.

### 2.5. Degree of Hydrolysis (DH)

OPA method was employed following the procedure of Church et al. [27] and Salami et al. [28] with minor modifications to measure DH at various hydrolysis time. To prepare OPA reagent, three solutions, A, B and C, were prepared separately. Solution A comprised of 7.62 g of sodium tetrahydroborate and 200 mg of sodium dodecyl sulphate (SDS) dissolved in 150 mL deionised water; Solution B consisted of 160 mg of OPA dissolved in 4 mL of ethanol 96%; and Solution C consisted of 400 μL of β-mercaptoethanol, in 50 mL deionised water. Mixing the 3 solutions would yield fresh OPA reagent. Then, 36 μL of sample (protein concentration = 20 mg/mL) was mixed with 270 μL of OPA reagent in a 96-well plate, incubated for 2 min at room temperature, and the absorbance was measured at 340 nm by employing a microplate reader system (model: PowerWave X340, Biotek Instruments Inc., Winooski, VT, USA). The following equation was employed to calculate the degree of hydrolysis according to Adler-Nissen [29]:

DH (%) = {(A_sample_ − A_protein_)/A_total_} × 100 (6)

in which, A_sample_ represents the sample absorbance after hydrolysis, A_protein_ signifies the sample absorbance before hydrolysis (negative control) and A_total_ denotes the absorbance of total protein after 24 h complete hydrolysis using 6M HCl.

### 2.6. Angiotensin Converting Enzyme (Ace) Inhibitory Activity Assay

ACE–inhibitory was determined according to Cushman and Cheung [30] and Ferreira et al. [31] with certain modifications. The assay was performed by first mixing 10 μL of sample (protein concentration = 20 mg/mL) and 10 μL of ACE (100 mU/mL) then incubated for 10 min, followed by addition of 50 μL 0.1 M sodium borate buffer (pH 8.3) comprising of 5 mM HHL and 0.3 M NaCl into the incubated sample. For control and blank, 10 μL and 20 μL of distilled water were respectively added. After incubation for 60 min at 37 °C, the reaction was terminated by introducing 75 μL of 1 M HCl. After the reaction was stopped, addition of 150 μL of pyridine followed by 75 uL of benzene sulphonyl chloride (BSC) was done. Then, the solution was vortexed for 1 min and cooled in ice bath. After cooling, 200 μL of the solution was transferred to a 96-well plate, and the absorbance was measured at 410 nm. ACE inhibition was calculated as follow:

ACE inhibition (%) = {(*C* − *S*)/(*C* − *B*)} × 100 (7)

where, *C* is the absorbance of control (ACE + substrate), *S* is the absorbance of sample (peptide + ACE + substrate) and *B* is the absorbance of blank (only substrate).

### 2.7. Determination of Antioxidant Activity

#### 2.7.1. 1, 1-diphenyl-2-picrylhydrazyl Free Radical Scavenging Assay

The percentage for 1, 1-diphenyl-2-picrylhydrazyl (DPPH•) free radical scavenging activity was obtained according to the method of Hwang et al. [32] with some modification. Briefly, 50 μL of WPC hydrolysate (WPCH, protein concentration = 20 mg/mL) was added to100 μL of DPPH• (0.15 mM, 100% methanol) and 50 μL deionised water. At room temperature, the mixture was placed in a dark room for 45 min and then the absorbance was measured at 517 nm. The scavenging ability of WPCH was calculated as follow:

DPPH• radical scavenging activity (%) = {(A_control_ − A_sample_)/A_control_} × 100 (8)

whereby, A_control_ represents the absorbance of control and A_sample_ denotes the absorbance of samples at 517 nm.

#### 2.7.2. Ferrous Ion Chelating Activity Assay

The ferrous ion chelating activity of WPCH was measured based on a previously defined method put forward by Dinis, Madeira and Almeida [33] with some changes. WPCH (100 μL) of concentrations between 0.005 mg/mL to 10 mg/mL, was mixed with 185 μL of double distilled water and 5 μL of 2 mmol/L iron dichloride solution, followed by addition of 10 μL of 5 mmol/L ferrozine solution and mixed in a vigorous manner. At room temperature, the mixture was incubated for 10 min, prior to absorbance measurement at 562 nm.

The following equation was employed to determine the chelating effect:

Metal chelating activity (%) = {(A_control_ − A_sample_)/A_control_} × 100 (9)

where, A_control_ represents the absorbance of control and A_sample_ denotes the absorbance of samples at 562 nm.

## 3. Results and Discussion

### 3.1. Proximate Analysis

Table 1 shows the proximate composition of WPC with the values of 5.64%, 76.13%, 3.91% and 2.96% for moisture, protein, fat and ash, respectively. Except for moisture and carbohydrate, all values remained comparable to that reported by Adamson [3], Suthar, Jana and Balakrishnan [4] and Whetstine, Croissant and Drake [5]. In particular, the protein content of WPC is high. Therefore, WPC is considered a valuable low-cost protein source to generate hydrolysate (a mixture of bioactive peptides) with dual functionalities upon optimized hydrolysis.

### 3.2. Molecular Weight of WPC Hydrolysates

**Figure 1:**
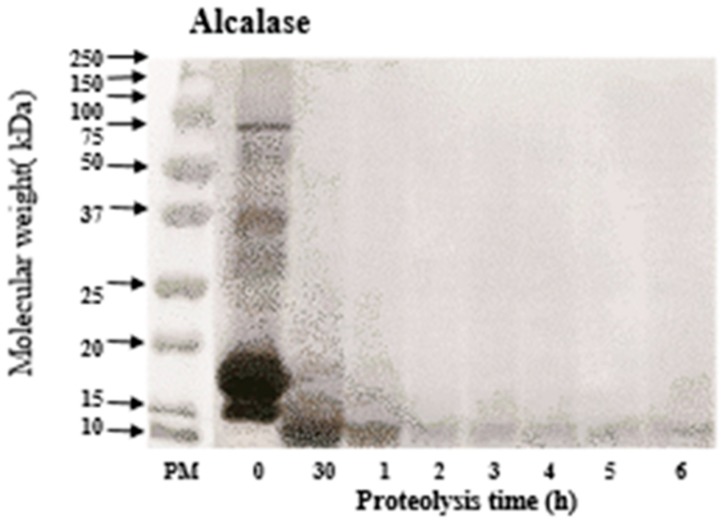
SDS-PAGE of native whey protein concentrate (WPC) and Alcalase-treated WPC at different hydrolysis hours.

The size of the generated peptides is essential for ACE inhibitory, antioxidative and other biological activities. SDS-PAGE was performed to investigate the molecular weight distribution of peptides from WPC hydrolysate at different hydrolysis times. Figure 1 shows that Alcalase efficiently degrades major WPC proteins, i.e., β-lactoglobulin and α-lactalbumin (M_w_: 15–20 kDa), into peptides of smaller fragments (below 10 kDa) after 30 min of hydrolysis. Also, serum albumin with heavier molecular weight (around 75 kDa), produces no visible band on the gel after 30 min. These observations are in line with the work performed by Pena-Ramos and Xiong [34], who reported a rapid degradation of WPC proteins into smaller peptides by protease A (an endopeptidase obtained from *Bacillus licheniformis*, now widely known as Alcalase).

### 3.3. Model Performance Appraisal on the Regression Elements

In terms of statistics, five parameters are crucial, which include estimated regression coefficient (*R*^2^), adjusted coefficient of determination (*R*^2^-adj), lack of fit values, Fisher test value (*f*-value) and regression *p*-values. The combination of high *R*^2^, a non-significant lack of fit, high *f*-value and low *p*-values signify a good fitness of experimental values to predicted values obtained from regression equations, and that the regression model is statistically sufficient to explain the experimental data. The *R*^2^-adj further improves the reliability of regression model by taking into consideration only the factors (x-values) that significantly affect the response (y-values).

Table 2 presents the predicted and experimental responses of DH (y_1_), ACE-inhibitory activity (y_2_), DPPH• (y_3_) and ferrous ion chelating (y_4_) under various hydrolysis conditions, at different combinations of temperature (x_1_), E/S ratio (x_2_), pH (x_3_) and time (x_4_). It can be seen that most experimental values were similar to predicted values. From Table 3, the values for *R*^2^ were 91.3% (y_1_), 90.0% (y_2_), 86.8% (y_3_) and 90.8% (y_4_) while the result for *R*^2^-adj were 87.4%, 83.7%, 71.4% and 80.1% for y_1_, y_2_, y_3_ and y_4_. The lack of fit values for y_1_, y_2_, y_3_ and y_4_ were 0.665, 0.060, 0.013 and 0.027 while the *f*-test values of y_1_, y_2_, y_3_ and y_4_ were 23.54, 14.33, 9.80 and 12.33, respectively. These results confirmed the validity of selected regression model to predict the responses (y-values).

### 3.4. Effects of Temperature, E/S Ratio, pH and Time on Different Responses

#### 3.4.1. Degree of Hydrolysis (DH)

From Table 2, DH between 17.9–93.3% was recorded for hydrolysis under different combinations of temperature, E/S ratio, pH and time. High DH (above 80%) was observed for 7 runs, out of a total of 27 runs, and this is supported by the work of See, Hoo and Babji [35] who reported a DH of 77.03% for fish skin hydrolysis that used a single enzyme of Alcalase. High DH indicates that the selected combinations of temperature, E/S ratio, pH and time were optimum to perform hydrolysis on large, bulky whey protein molecules to effectively cut them into smaller peptides.

**Figure 2:**
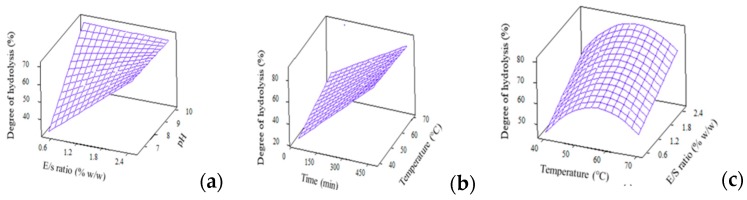
3D surface plots for degree of hydrolysis (%) as affected by (**a**) E/S ratio and pH; (**b**) Time and temperature and (**c**) Temperature and E/S ratio.

The analysis of variance (*p* < 0.05) and regression coefficients for DH are depicted in Table 3. As shown, the regression model for DH, when expressed in full quadratic equation, is significant at *p* = 0.000. This confirms the adequacy of model to fit the DH experimental data. The squares effects were identified as temp*temp and time*time while the interaction effects were found to be temp*pH and E/S*pH. The high *R*^2^, non-significant lack-of-fit and large *f*-value implies a good fit of experimental values to predicted model. Figure 2 depicts the interaction effects from independent factors, comprised of temperature, E/S ratio and pH, which contributed to the changes in DH. From Figure 2c, DH increased with hydrolysis temperature, reached a maximum point then decreased following higher temperature, forming a quadratic surface plot. This is due to enzyme denaturation of Alcalase at high temperatures which reduces its efficiency to hydrolyse WPC molecules into smaller peptide fragments, and thus results in lower DH values.

#### 3.4.2. ACE-inhibitory Activity

**Figure 3:**
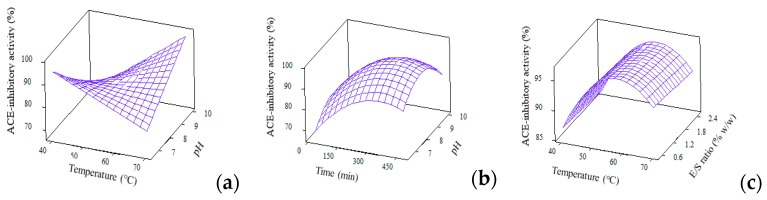
3D surface plots for ACE-inhibitory activity (%) as affected by (**a**) Temperature and pH; (**b**) Time and pH and (**c**) Temperature and E/S ratio.

The analysis of variance (*p* < 0.05) and regression coefficients for ACE-inhibitory activity were generated from reduced full quadratic model and were compiled in Table 3. Six terms were found to be significant to explain the changes in ACE-inhibitory activity, namely, temp*temp, pH*pH, time*time, temp*pH, temp*time and E/S*pH. The regression model for ACE-inhibitory activity with high *R*^2^ of 89.96%, nonsignificant lack-of-fit of 0.06 and large f-value of 14.33 denotes the goodness of fit of experimental results to the predicted ones. The surface plots for ACE-inhibitory activity were constructed based on the interaction effects of temperature, E/S ratio, pH and time (Figure 3).

ACE-inhibitory activity of WPCHs was highly affected by hydrolysis parameters as well as the resulting DH. At higher DH, a higher ACE inhibitory bioactivity was achieved. This result is in line with the work reported by Silvestre et al. [36] and Ghanbari et al. [37], in which the ACE-inhibitory activity of protein hydrolysates was affected by the type of enzymes as well as the degree of hydrolysis. Also, the increased level of E/S ratio and alkali pH resulted in high values of DH to indicate the release of potent peptides which further contributed towards the strong ACE-inhibitory activity. These findings are comparable to another work reported by van der Ven et al. [38] which used the same sample of whey protein hydrolysate.

#### 3.4.3. Antioxidant Activities

**Figure 4:**
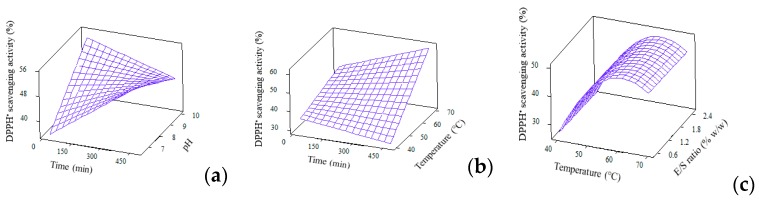
3D surface plots for DPPH• radical scavenging (%) as affected by (**a**) Time and pH; (**b**) Time and temperature and (**c**) Temperature and E/S ratio.

Unlike typical works which perform only single antioxidant assay in RSM study, current work reported two antioxidant activities, i.e., DPPH• radical scavenging and ferrous chelating activities. The reduced quadratic model of DPPH• radical scavenging is shown in Table 3. Even though this model contains only two significant terms, contributed by temp*temp and pH*time, the overall regression equation is significant (*p* = 0.000) due to the strong effect from linear, squares and interaction. Figure 4 presents the 3D plots regarding DPPH• radical scavenging activity. Similar to the effect of temperature on DH, as previously discussed in Section 3.4, the effect of temperature on DPPH• radical scavenging activity is also prominent. As the hydrolysis temperature increased, the scavenging activity increased until a maximum point, then decreased with higher temperature. This is, again, due to the application of temperature which allow/deter optimum Alcalase activity to generate peptides with DPPH• radical scavenging activity. The ability for scavenging free radicals could be attributed to the peptides’ ability to donate hydrogen, neutralise or stabilise free radicals and then cease their propagation. This potency is strongly affected by the amino acid composition in samples, especially aromatic amino acids like tryptophan, phenylalanine, histidine and tyrosine as well as hydrophobic amino acids like alanine, leucine and valine, with methionine playing the most critical role on affecting the scavenging activity [39,40,41].

**Figure 5:**
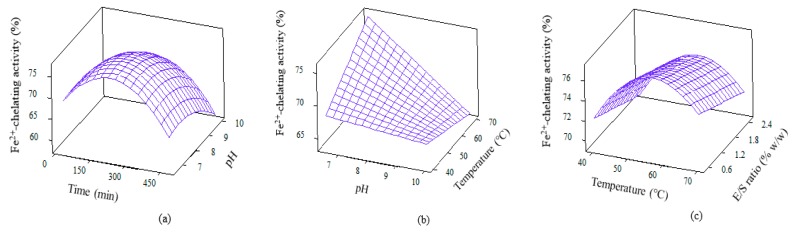
3D surface plots for ferrous chelating activity as affected by (**a**) Time and pH; (**b**) pH and temperature and (**c**) Temperature and E/S ratio.

Ferrous chelating activity, which helps to minimise lipid oxidation, was exhibited by WPC hydrolysate. The quadratic model for this bioactivity is depicted in Table 3. The model is significant (*p* = 0.000) and encompasses all linear, squares, interaction effects with *p* values of 0.000, 0.000 and 0.024, respectively. Four significant terms were reported for ferrous chelating activity, which consisted of mainly squares effect, i.e., temp*temp, pH*pH, time*time, along with an interaction effect of E/S*time. The 3D plots for ferrous chelating activity are shown in Figure 5. The potency of peptides are thought to be affected by sources of protein, type of proteases, amino acid composition and time of hydrolysis [42,43]. The higher ferrous ion chelating activity demonstrated by WPCH proves the suitability of Alcalase as the choice of enzyme to produce biopeptide molecules.

### 3.5. Optimized Hydrolysis Condition For WPC and Model Validation

**Figure 6:**
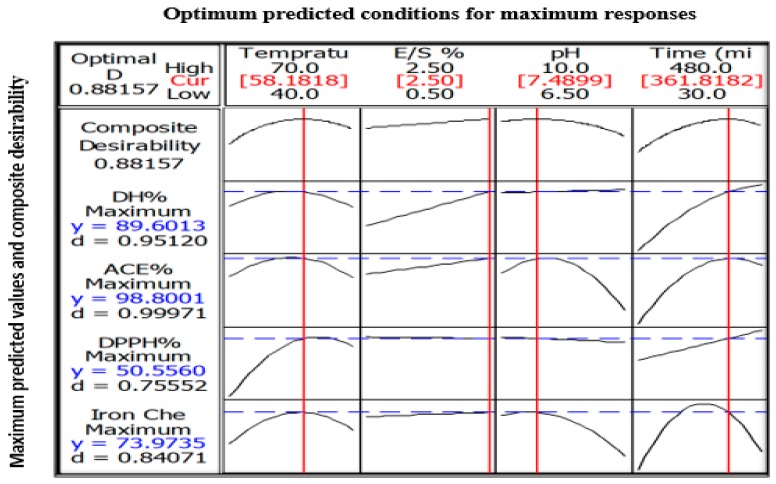
The predicted maximum responses (DH, ACE-inhibitory, DPPH• radical scavenging, ferrous ion chelating activity) based on predicted optimum hydrolysis parameters (temperature, E/S ratio, pH and hydrolysis time). Composite desirability = overall desirability encompassing all responses and d = individual desirability. Optimum hydrolysis parameters are denoted as red lines while the maximum responses are denoted as blue dotted lines.

**Figure 7:**
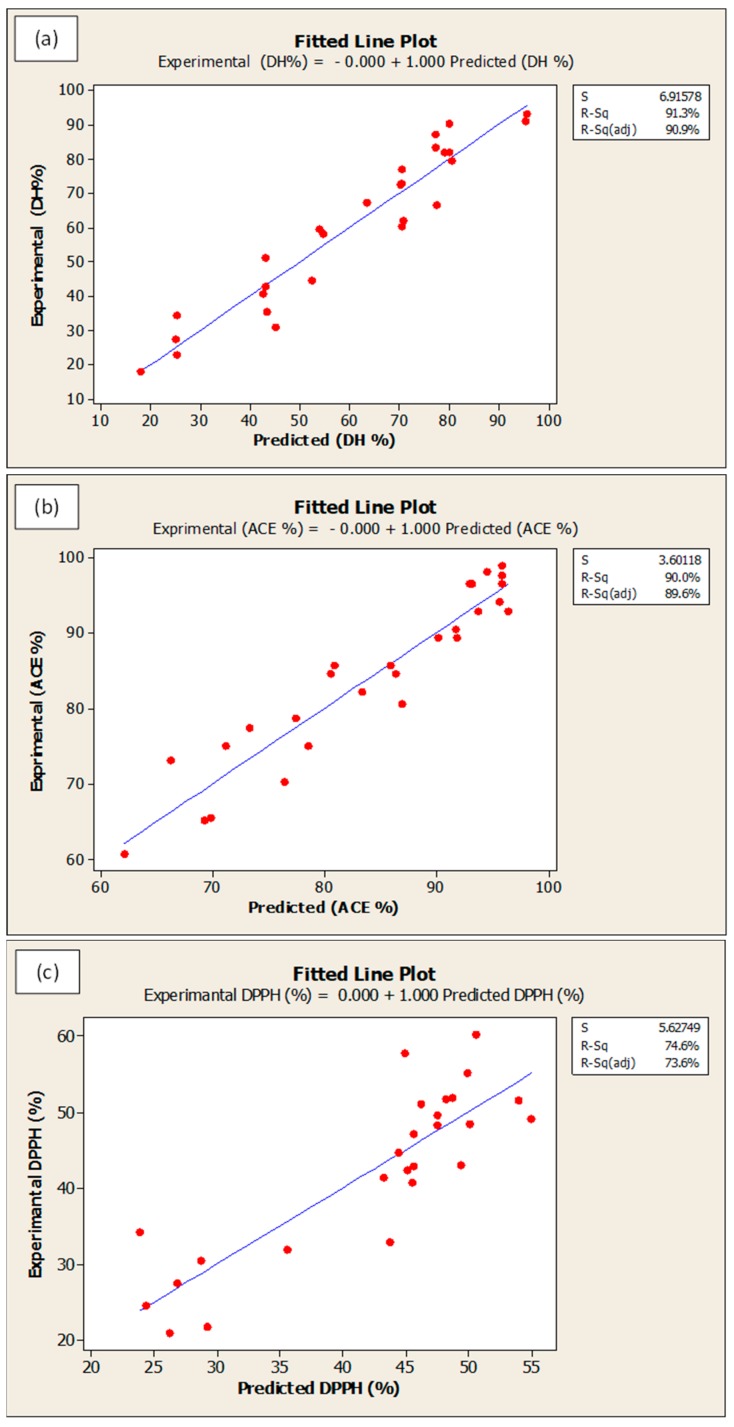

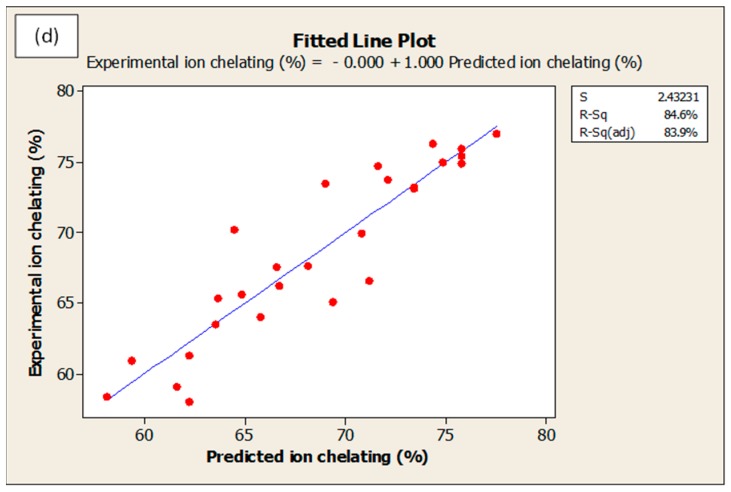
Fitted line plots for predicted and experimental values for (**a**) degree of hydrolysis, (**b**) ACE-inhibition, (**c**) DPPH• radical scavenging activity and (**d**) ferrous ion chelating activity. All values are reported in %.

Optimization procedure was performed using Box-Behnken design. Figure 6 presents the optimum predicted hydrolysis conditions for WPC that could theoretically produces the maximum DH, ACE-inhibitory, DPPH• radical scavenging and ferrous ion chelating activities. The predicted optimal conditions were as follow: temperature = 58.2 °C, E/S ratio = 2.5%, pH = 7.5, and hydrolysis time = 361.8 min. The predicted parameters were employed in actual experiments for model validation purpose. The model was confirmed to be validate via two observations: (i) The overall desirability (D-value) of 0.88 indicated a high level of confidence for the model to produce the responses as predicted; and (ii) The experimental values for DH (89.2% vs. theoretical 89.6%), ACE-inhibitory activity (98.4% vs. theoretical 98.8%), DPPH• radical scavenging (50.1% vs. theoretical 50.6%) and ferrous ion chelating (73.1% vs. theoretical 74.0%) were identical to their respective predicted values. On the other hand, the goodness-of-fit between experimental and predicted data is depicted in Figure 7a–d. In all figures, the data points fall closely to the regression line. Also, high R^2^ of 91.3%, 90.0%, 74.6% and 84.6% for DH, ACE inhibitory, DPPH• scavenging and ferrous ion chelating, respectively, indicated that most of the variabilities of these responses were explained by their linear models.

## 4. Conclusions

This study represents a pioneer work on the optimization of whey protein concentrate hydrolysis using Alcalase to generate biopeptides with dual functionalities (ACE inhibition and antioxidant activities). The optimized hydrolysis parameters were determined as follow: Temperature = 58.2 °C, E/S ratio = 2.5%, pH = 7.5 and hydrolysis time = 361.8 min. Under such conditions, the experimental values did not show any statistical differences from the predicted values but fitted closely to them. Results showed that the selected hydrolysis parameters were able to produce whey protein hydrolysate with dual bioactivities of ACE inhibition and antioxidant at desirable levels. This study proved the reliability of the selected regression models to sufficiently explain the factor-response relationship during WPC hydrolysis and that the predicted optimum hydrolysis conditions are valid to generate, from WPC, bioactive peptides with ACE inhibitory and antioxidative activities. Further work on the in vivo efficacy and subsequent clinical trials of WPC hydrolysate is deemed appropriate.

## Figures and Tables

**Table 1:** Comparison of proximate composition of whey protein concentrate with literature. All values are reported in dry basis (%).

Parameters	Present Study (Mean ± sd)	Literature Data *
Moisture (%)	5.64 ± 0.03	3.8–4.5
Protein (%)	76.13 ± 0.23	74.8–80.0
Crude fat (%)	3.91 ± 0.75	1.8–7.2
Ash (%)	2.96 ± 0.01	2.7–7.5
Carbohydrates (%)	11.93 ± 0.00	3.5–8.9

* Reported data were compiled from three literatures: Adamson [3] Suthar et al. [4] and Whetstine et al. [5]. sd = standard deviation from 3 readings.

**Table 2:** Behnken design, predicted and experimental values of four response variables (y_1_, y_2_, y_3_, y_4_) as affected by temperature (°C), E/S ratio (%), pH and hydrolysis time (min).

Run Order	x_1_	x_2_	x_3_	x_4_	% DH (y_1_)		%ACE Inhibition (y_2_)		% DPPH• (y_3_)		% Ferrous Ion Chelating (y_4_)	
					Experimental	Predicted	Experimental	Predicted	Experimental	Predicted	Experimental	Predicted
1	40	0.5	8.25	255	30.71	45.22	27.53	26.85	27.53	26.85	73.78	72.09
2	70	0.5	8.25	255	44.63	52.36	51.10	46.28	51.10	46.28	76.30	74.33
3	40	2.5	8.25	255	67.42	63.67	20.97	26.23	20.97	26.23	65.04	69.37
4	70	2.5	8.25	255	62.09	70.81	47.13	45.65	47.13	45.65	74.70	71.61
5	55	1.5	6.5	30	22.67	25.22	31.87	35.59	31.87	35.59	73.48	68.97
6	55	1.5	10	30	42.64	43.29	51.47	54.03	51.47	54.03	58.00	62.23
7	55	1.5	6.5	480	87.35	77.50	49.10	55.03	49.10	55.03	65.63	64.82
8	55	1.5	10	480	91.07	95.57	40.77	45.53	40.77	45.53	58.37	58.08
9	40	1.5	8.25	30	17.90	18.09	34.20	23.81	34.20	23.81	63.44	63.51
10	70	1.5	8.25	30	34.46	25.23	41.33	43.23	41.33	43.23	64.04	65.75
11	40	1.5	8.25	480	72.37	70.37	21.67	29.27	21.67	29.27	60.93	59.36
12	70	1.5	8.25	480	66.52	77.52	51.77	48.7	51.77	48.7	59.00	61.60
13	55	0.5	6.5	255	40.58	42.54	42.80	45.62	42.8	45.62	77.00	77.55
14	55	2.5	6.5	255	79.65	80.60	57.73	45.00	57.73	45.00	74.96	74.83
15	55	0.5	10	255	82.06	80.22	48.33	50.09	48.33	50.09	69.96	70.81
16	55	2.5	10	255	81.91	79.06	42.93	49.46	42.93	49.46	67.59	68.09
17	40	1.5	6.5	255	58.15	54.87	24.5	24.31	24.5	24.31	66.56	71.16
18	70	1.5	6.5	255	51.01	43.09	32.8	43.73	32.8	43.73	73.11	73.40
19	40	1.5	10	255	59.72	54.02	30.4	28.77	30.4	28.77	70.19	64.42
20	70	1.5	10	255	90.43	80.08	51.70	48.20	51.70	48.20	66.22	66.66
21	55	0.5	8.25	30	27.44	25.03	42.37	45.12	42.37	45.12	73.22	73.43
22	55	2.5	8.25	30	35.25	43.48	44.57	44.50	44.57	44.50	65.37	63.64
23	55	0.5	8.25	480	83.48	77.31	60.13	50.59	60.13	50.59	61.22	62.21
24	55	2.5	8.25	480	93.28	95.76	55.17	49.96	55.17	49.96	67.51	66.56
25	55	1.5	8.25	255	60.21	70.61	48.17	47.54	48.17	47.54	75.93	75.76
26	55	1.5	8.25	255	76.95	70.61	49.53	47.54	49.53	47.54	74.93	75.76
27	55	1.5	8.25	255	72.94	70.61	48.30	47.54	48.30	47.54	75.41	75.76

Remark: Independent variables: x_1_ = Temperature; x_2_ = E/S ratio; x_3_ = pH; x_4_ = hydrolysis time.

**Table 3:** Of variance at *p* < 0.05 and regression coefficients for DH (y_1_), ACE inhibition (y_2_), DPPH• (y_3_) and ferrous ion chelating activity (y_4_), expressed in full quadratic models.

Factors	y_1_	*p*-Value	y_2_	*p*-Value	y_3_	*p*-Value	y_4_	*p*-Value
Regression		0.000		0.000		0.000		0.000
Linear		0.000		0.004		0.000		0.000
Squares		0.001		0.000		0.000		0.000
Interaction		0.013		0.000		0.038		0.024
Constant	−116.761	0.203	−57.8367	0.408	−190.337	0.000	−31.8992	0.355
x_1_	3.421	0.110	−0.9430	0.428	6.167	0.000	1.9866	0.003
x_2_	55.453	0.010	33.1444	0.007	−0.313	0.865	−5.3641	0.009
x_3_	−6.253	0.511	34.2940	0.007	5.799	0.020	13.9083	0.041
x_4_	0.219	0.000	0.0542	0.210	0.158	0.027	0.0609	0.001
x_1_*x_1_	−0.056	0.001	−0.0286	0.003	−0.050	0.000	−0.0174	0.004
x_2_*x_2_		NS		NS		NS		NS
x_3_*x_3_		NS	−3.3472	0.000		NS	−0.9596	0.022
x_4_*x_4_	0.000	0.005	−0.0002	0.000		NS	−0.0002	0.000
x_1_*x_2_		NS		NS		NS		NS
x_1_*x_3_	0.360	0.032	0.4761	0.000		NS		NS
x_1_*x_4_		NS	0.0014	0.050		NS		NS
x_2_*x_3_	−5.603	0.027	−4.0816	0.006		NS		NS
x_2_*x_4_		NS		NS		NS	0.0157	0.024
x_3_*x_4_		NS		NS	−0.018	0.038		NS
*R* ^2^	91.27%		89.96%		74.62%		84.56%	
*R*^2^-adj	87.40%		83.68%		67.01%		77.70%	
Lack of fit		0.665		0.060		0.013		0.027
*f*-value	23.54		14.33		9.80		12.33	

NS means not significant (*p* > 0.05) and thus the respective regression coefficient is not reported.

## References

[1] Zhou B., Bentham J., Di Cesare M., Bixby H., Danaei G., Cowan M.J., Paciorek C.J., Singh G., Hajifathalian K., Bennett J.E. (2017). Worldwide trends in blood pressure from 1975 to 2015: A pooled analysis of 1479 population-based measurement studies with 19 1 million participants. Lancet.

[2] Kearney P.M., Whelton M., Reynolds K., Muntner P., Whelton P.K., He J. (2005). Global burden of hypertension: Analysis of worldwide data. Lancet.

[3] Adamson N., Bylund G. (2015). Whey processing. Dairy Processing Handbook.

[4] Suthar J., Jana A., Balakrishnan S. (2017). High protein milk ingredients-A tool for value-addition to dairy and food products. J. Dairy Vet. Anim. Res..

[5] Whetstine M.C., Croissant A., Drake M. (2005). Characterization of dried whey protein concentrate and isolate flavor. J. Dairy Sci..

[6] del Mar Contreras M., Hernández-Ledesma B., Amigo L., Martín-Álvarez P.J., Recio I. (2011). Production of antioxidant hydrolyzates from a whey protein concentrate with thermolysin: Optimization by response surface methodology. LWT Food Sci. Technol..

[7] Muro Urista C., Álvarez Fernández R., Riera Rodriguez F., Arana Cuenca A., Tellez Jurado A. (2011). Production and functionality of active peptides from milk. Food Sci. Technol. Int..

[8] Lin S., Tian W., Li H., Cao J., Jiang W. (2012). Improving antioxidant activities of whey protein hydrolysates obtained by thermal preheat treatment of pepsin, trypsin, alcalase and flavourzyme. Int. J. Food Sci. Technol..

[9] Guo Y., Pan D., Tanokura M. (2009). Optimisation of hydrolysis conditions for the production of the angiotensin-I converting enzyme (ACE) inhibitory peptides from whey protein using response surface methodology. Food Chem..

[10] Morais H.A., Silvestre M.P.C., Silva M.R., Silva V.D.M., Batista M.A., e Silva A.C.S., Silveira J.N. (2015). Enzymatic hydrolysis of whey protein concentrate: Effect of enzyme type and enzyme: Substrate ratio on peptide profile. J. Food Sci. Technol..

[11] Tavares T., Malcata F., Hernández-Ledesma B. (2013). Whey proteins as source of bioactive peptides against hypertension. Bioactive Food Peptides in Health and Disease.

[12] Wu Q., Zhang X., Jia J., Kuang C., Yang H. (2018). Effect of ultrasonic pretreatment on whey protein hydrolysis by alcalase: Thermodynamic parameters, physicochemical properties and bioactivities. Process Biochem..

[13] Silveira S.T., Martínez Maqueda D., Recio I., Hernández Ledesma B. (2013). Dipeptidyl peptidase-IV inhibitory peptides generated by tryptic hydrolysis of a whey protein concentrate rich in β-lactoglobulin. Food Chem..

[14] Boyacı D., Korel F., Yemenicioğlu A. (2016). Development of activate-at-home-type edible antimicrobial films: An example pH-triggering mechanism formed for smoked salmon slices using lysozyme in whey protein films. Food Hydrocoll..

[15] Madureira A., Tavares T., Gomes A.M.P., Pintado M., Malcata F.X. (2010). Invited review: Physiological properties of bioactive peptides obtained from whey proteins. J. Dairy Sci..

[16] O’keeffe M.B., Conesa C., FitzGerald R.J. (2017). Identification of angiotensin converting enzyme inhibitory and antioxidant peptides in a whey protein concentrate hydrolysate produced at semi-pilot scale. Int. J. Food Sci. Technol..

[17] Ajlouni S., Pan Y. (2014). Effect of pH and whey protein isolate to glucose ratios on the formation of Maillard reaction products as antioxidants. Kasetsart J. (Nat. Sci.).

[18] Zhang Q.X., Wu H., Ling Y.F., Lu R.R. (2013). Isolation and identification of antioxidant peptides derived from whey protein enzymatic hydrolysate by consecutive chromatography and Q-TOF MS. J. Dairy Res..

[19] Naik L., Mann B., Bajaj R., Sangwan R., Sharma R. (2013). Process optimization for the production of bio-functional whey protein hydrolysates: Adopting response surface methodology. Int. J. Pept. Res..

[20] Athira S., Mann B., Saini P., Sharma R., Kumar R., Singh A.K. (2015). Production and characterisation of whey protein hydrolysate having antioxidant activity from cheese whey. J. Sci. Food Agric..

[21] Fenoglio C., Vierling N., Manzo R., Ceruti R., Sihufe G., Mammarella E. (2016). Whey protein hydrolysis with free and immobilized alcalase^®^: Effects of operating parameters on the modulation of peptide profiles obtained. Am. J. Food Technol..

[22] Bezerra M., Santelli R., Oliveira E., Villar L., Escaleira L. (2008). Response surface methodology (RSM) as a tool for optimization in analytical chemistry. Talanta.

[23] AOAC International (2005). Official Methods of Analysis of AOAC International.

[24] Laemmli U.K. (1970). Cleavage of structural proteins during the assembly of the head of bacteriophage T4. Nature.

[25] Alexopoulos E. (2010). Introduction to multivariate regression analysis. Hippokratia.

[26] Mayer R., Montgomery D. (1995). Response Surface Methodology: Process and Product Optimization Using Designed Experiments.

[27] Church F., Swaisgood H., Porter D., Catignani G. (1983). Spectrophotometric assay using o-phthaldialdehyde for determination of proteolysis in milk and isolated milk proteins. J. Dairy Sci..

[28] Salami M., Yousefi R., Ehsani M.R., Dalgalarrondo M., Chobert J.M., Haertlé T., Razavi S.H., Saboury A.A., Niasari-Naslaji A., Moosavi-Movahedi A.A. (2008). Kinetic characterization of hydrolysis of camel and bovine milk proteins by pancreatic enzymes. Int. Dairy J..

[29] Adler-Nissen J. (1986). Enzymic Hydrolysis of Food Proteins.

[30] Cushman D., Cheung H. (1971). Spectrophotometric assay and properties of the angiotensin-converting enzyme of rabbit lung. Biochem. Pharm..

[31] Ferreira I., Pinho O., Mota M., Tavares P., Pereira A., Goncalves M., Torres D., Rocha C., Teixeira J. (2007). Preparation of ingredients containing an ACE-inhibitory peptide by tryptic hydrolysis of whey protein concentrates. Int. Dairy J..

[32] Hwang J.Y., Shyu Y.S., Wang Y.T., Hsu C.K. (2010). Antioxidative properties of protein hydrolysate from defatted peanut kernels treated with esperase. LWT Food Sci. Technol..

[33] Dinis T.C., Madeira V.M., Almeida L.M. (1994). Action of phenolic derivatives (acetaminophen, salicylate, and 5-aminosalicylate) as inhibitors of membrane lipid peroxidation and as peroxyl radical scavengers. Arch. Biochem. Biophys..

[34] Pena-Ramos E., Xiong Y. (2001). Antioxidative activity of whey protein hydrolysates in a liposomal system. J. Dairy Sci..

[35] See S.F., Hoo L.L., Babji A.S. (2011). Optimization of enzymatic hydrolysis of Salmon (*Salmo salar*) skin by Alcalase. Int. Food Res. J..

[36] Silvestre M.P.C., Silva M.R., Silva V.D.M., Souza M.W.S., Junior L., Oliveira C., Afonso W.O. (2012). Analysis of whey protein hydrolysates: Peptide profile and ACE inhibitory activity. Braz. J. Pharm. Sci..

[37] Ghanbari R., Zarei M., Ebrahimpour A., Abdul-Hamid A., Ismail A., Saari N. (2015). Angiotensin-I converting enzyme (ACE) inhibitory and anti-oxidant activities of sea cucumber (*Actinopyga lecanora*) hydrolysates. Int. J. Mol. Sci..

[38] van der Ven C., Gruppen H., de Bont D., Voragen A. (2002). Optimisation of the angiotensin converting enzyme inhibition by whey protein hydrolysates using response surface methodology. Int. Dairy J..

[39] Chi C.F., Hu F.Y., Wang B., Li Z.R., Luo H.Y. (2015). Influence of amino acid compositions and peptide profiles on antioxidant capacities of two protein hydrolysates from skipjack tuna (*Katsuwonus pelamis*) dark muscle. Mar. Drugs.

[40] Luo S., Levine R.L. (2009). Methionine in proteins defends against oxidative stress. FASEB J..

[41] Zou T.B., He T.P., Li H.B., Tang H.W., Xia E.Q. (2016). The structure-activity relationship of the antioxidant peptides from natural proteins. Molecules.

[42] Karamać M., Kosińska-Cagnazzo A., Kulczyk A. (2016). Use of different proteases to obtain flaxseed protein hydrolysates with antioxidant activity. Int. J. Mol. Sci..

[43] O’Loughlin I.B., Kelly P.M., Murray B.A., FitzGerald R.J., Brodkorb A. (2015). Molecular characterization of whey protein hydrolysate fractions with ferrous chelating and enhanced iron solubility capabilities. J. Agric. Food Chem..

